# The Role of Immune Cells in the Pathogenesis of Idiopathic Pulmonary Fibrosis

**DOI:** 10.3390/medicina59111984

**Published:** 2023-11-10

**Authors:** Yahan Xu, Peixiang Lan, Tao Wang

**Affiliations:** 1 Department of Respiratory and Critical Care Medicine, Tongji Hospital, Tongji Medical College, Huazhong University of Science and Technology, Wuhan 430030, China; m202176117@hust.edu.cn; 2 The Center for Biomedical Research, National Health Committee (NHC) Key Laboratory of Respiratory Disease, Tongji Hospital, Tongji Medical College, Huazhong University of Science and Technology, Wuhan 430030, China; 3 Institute of Organ Transplantation, Tongji Hospital, Tongji Medical College, Huazhong University of Science and Technology, Wuhan 430030, China; 4 Key Laboratory of Organ Transplantation, Ministry of Education; NHC Key Laboratory of Organ Transplantation; Key Laboratory of Organ Transplantation, Chinese Academy of Medical Sciences, Wuhan 430030, China

**Keywords:** idiopathic pulmonary fibrosis, immune cell, macrophage, T cells

## Abstract

Idiopathic pulmonary fibrosis (IPF) is a devastating disease of unknown etiology with limited treatment options. The role of the immune system in IPF has received increasing attention. Uncontrolled immune responses drive the onset and progression of IPF. This article provides an overview of the role of innate immune cells (including macrophages, neutrophils, mast cells, eosinophils, dendritic cells, nature killer cells, nature kill cells and γδ T cells) and adaptive immune cells (including Th1 cells, Th2 cells, Th9 cells, Th17 cells, Th22 cells, cytotoxic T cells, B lymphocytes and Treg cells) in IPF. In addition, we review the current status of pharmacological treatments for IPF and new developments in immunotherapy. A deeper comprehension of the immune system’s function in IPF may contribute to the development of targeted immunomodulatory therapies that can alter the course of the disease.

## 1. Introduction

Idiopathic pulmonary fibrosis (IPF) is a chronic, progressive, fibrotic interstitial lung disease that predominantly affects older adults [1,2]. IPF is mostly disseminated worldwide, with an estimated global incidence of over 60 cases per 100,000 individuals [3]. The median survival period for patients following diagnosis is just 2–3 years [4], with a mortality rate higher than that of most tumors. In patients with IPF, fibroblasts undergo extensive proliferation and release of the extracellular matrix (ECM) in the healthy tissue, destroying the alveolar structure and reducing lung compliance. The gas exchange function of the lung is disrupted, which eventually leads to respiratory failure and death [5].

The etiology of IPF is unknown and its pathogenesis has not been fully elucidated. Possible causative factors, such as aging, genetic differences, living environment and epigenetic reprogramming, can induce the development of the disease in individuals. There is increasing evidence to support the idea that an abnormality in the pulmonary immune system is an important factor that contributes to IPF. Immune cells play roles in the immune response as well as in the inflammatory response, and they secrete a large number of cytokines and chemokines [6]. In this review, we briefly discuss the potential mechanisms of immune cells in IPF.

## 2. The Role of Innate Immune Cells in IPF

### 2.1. Macrophages

Macrophages are innate immune cells that perform phagocytosis and eliminate pathogens as part of physiological processes [7]. Macrophages are abundantly present in the lung microenvironment, where they are mainly found as alveolar macrophages (AMs) and interstitial macrophages (IMs) [8]. Circulating monocytes are known to act as a new source of macrophages when AMs are damaged [9,10]. Due to the degree of polarization, the surrounding microenvironment and the severity of fibrosis, AMs have a double-sided effect that both promotes and inhibits fibrosis [11]. On the one hand, AMs can reduce collagen by secreting matrix metalloproteinases (MMPs) to break down the ECM or directly uptake collagen to reduce ECM deposition [12]. On the other hand, AMs can also secrete various pro-fibrotic cytokines and chemokines, thus contributing to the development of pulmonary fibrosis [13,14]. AMs can be polarized into M1 and M2 phenotype macrophages, whereby M1-type macrophages play a pivotal role in the pro-inflammatory host defense reactions, and M2-type macrophages contribute to anti-inflammatory responses and tissue remodeling [15,16]. M2 macrophages are integral players in tissue repair, secreting inflammatory inhibitory factors and promoting ECM production by epithelial cells. Cumulative evidence indicates that the M2 phenotype, as opposed to the M1 phenotype, prevails in the lung during the progression of IPF [17,18,19]. M2 macrophage-secreted transforming growth factor-β (TGF-β) leads to the promotion of pulmonary fibrosis. However, the depletion of M2 macrophages leads to the amelioration of fibrosis [20].

Recent single-cell sequencing data have been used to characterize the macrophage diversity in human lungs from both deceased donors without lung disease and individuals with IPF who have received a lung transplant [21]. SPP1^+^ macrophages have been found to play a critical role in IPF [22]. Interestingly, lots of single-cell RNA sequencing analyses have shown that SPP1^+^ macrophages accumulate in fibrotic niches and promote fibrosis formation in organs such as the liver, heart and kidney. In lungs, these SPP1^+^ macrophages predominate in fibrotic niches, whereas the number of FABP4^+^ macrophages, which may be homeostatic tissue-resident alveolar macrophages with gene signatures FABP4, MRC1 and MARCO, is higher in healthy lungs and nonfibrotic regions [23]. Functional enrichment analysis with GO biological processes demonstrated that SPP1^+^ macrophages are enriched in processes associated with fibrosis [21]. Trajectory inference analyses have demonstrated that SPP1^+^ macrophages are derived from monocytes, which are then recruited into injury and disease regions via CCR2 and CCR5. SPP1^+^ macrophages localize adjacent to ECM-producing fibroblasts and epithelial cells and may reprogram these cells using cytokines and chemokines [24].

Interleukins (ILs) are known to induce the polarization of M2-type macrophages and are involved in the pathogenesis of pulmonary fibrosis [25]. Signal transduction and activator of transcription 6 (STAT6) is a transcription protein whose activation is associated with lung fibrosis [26]. IL-4 and IL-13 can directly activate STAT6-induced polarization of M2-type macrophages [27]. Additionally, IL-24 can inhibit the suppressor of cytokine signaling proteins (SOCS) including SOCS 1 and SOCS 3, and enhance STAT6/PPAR-γ signaling to promote M2-type macrophage polarization [27]. In the final stage of tissue injury and inflammation, macrophages are polarized into M2-type macrophages under the influence of multiple stimulatory factors. These cells participate in tissue repair and fibrotic disease development by inhibiting inflammation. M2-type macrophages can secrete a variety of growth factors and chemokines. TGF-β induces epithelial cell damage and apoptosis and directly activates fibroblasts to produce collagen [28]. It has been shown that TGF-β is a major contributor to tissue repair through the promotion of ECM deposition, fibroblast to myofibroblast differentiation and the epithelial–mesenchymal transition (EMT) [29]. After damage to the alveolar epithelium, M2-type macrophages localize near the site of injury and secrete the chemokine C-C motif ligand 18 (CCL18) to facilitate collagen production by fibroblasts [30].

### 2.2. Neutrophils

Neutrophils originate from the bone marrow and play an important role in the innate immune system. In the lungs, they can participate in tissue remodeling [31]. Neutrophils do not only have antimicrobial functions, but they also shape the tissue environment by releasing proteases, oxidative enzymes, cytokines, and chemokines [32].

Neutrophil elastase (NE) is one of the important neutrophil-derived toxic molecules. In a normal organism, NE is regulated by protease inhibitors. NE is involved in the cleavage of inflammatory mediators, as well as in the induction of cytokines and chemotactic factors [33]. NE hydrolyses bronchial tissue and degrades several ECM components, including collagen type I, laminin, fibronectin and elastin, thus damaging the alveolar epithelial cells and capillary endothelial cells and destroying the alveolar structure, leading to pulmonary fibrosis [34]. MMPs are a class of enzymes involved in tissue remodeling and ECM degradation. Initially, they were thought to have anti-fibrotic properties [35]. Neutrophils are mainly involved in the secretion of MMP-8 and MMP-9 [12]. In vivo, MMP-8 deficiency increased the activity of antifibrotic cytokine IL-10 and significantly improved lung fibrosis [36]. During pulmonary fibrosis, MMP-9 levels are mainly increased in neutrophils [37]. The level of MMP-9 in the sputum of patients with IPF correlates with their neutrophil levels [38]. Some studies have shown that neutrophils can also release a substance called neutrophil extracellular traps (NETs) to regulate the procession of innate immunity. NETs may play multiple roles in the early inflammatory process of pulmonary fibrosis. On the one hand, histones in NETs are cytotoxic components that induce lung epithelial cell death in a concentration-dependent manner and promote early inflammation [39]. On the other hand, aggregated NETs can degrade pro-inflammatory mediators to avoid excessive inflammation [40]. In addition to being involved in early inflammatory processes, NETs promote fibroblast differentiation [41]. Chronic elevation of this NET component can lead to lung injury and excess matrix formation, prompting age-related interstitial lung fibrosis [42].

### 2.3. Mast Cells

Mast cells originate from pluripotent progenitor cells and undergo expansion and maturation in the bone marrow. Mast cell maturation and differentiation are influenced by a variety of cytokines, including nerve growth factor (NGF) and colony-stimulating factor (STF) [43]. Mature mast cells are transported throughout the body via blood circulation and are distributed along the blood vessels. Mast cell membranes are interconnected with the basement membranes of vascular epithelial cells, and together form the perivascular microenvironment [44]. Mast cells are mainly classified into tryptase-only-positive mast cells (MCTs) and tryptase- and chymase-positive mast cells (MCTCs). MCTs are the predominant mast cell type in the lungs. They are distributed in the alveoli, airway epithelium and submucosa. MCTCs are predominantly found in connective tissues, but they are also located in the lungs [44].

Increasing evidence suggests that mast cells play an important role in tissue and organ fibrosis. Mast cells secrete a variety of cytokines and biologically active substances to induce fibroblast activation, and they synthesize collagen to participate in the fibrotic procession [45]. Chymotrypsin-like enzymes secreted by activated mast cells are important pro-fibrotic factors. Secondary lymph edema of the lower limb is often accompanied by dermal fibrosis, with large, abnormal aggregates of mast cells in the thickened dermis. The levels of both chymotrypsin-like and trypsin-like enzymes were found to be significantly higher in these pathological tissues [46]. Trypsin-like enzymes are proteases secreted by mast cells and are specific to mast cells. The abnormal proliferation of mast cells that synthesize trypsin-like enzymes has been detected in many lesions associated with fibrosis, such as chronic submandibular inflammation, fibrous papules, oral fibromas [47], scar formation [48] drug-induced fiber formation [49], etc. Further, TGF-β is a major regulator of fibrosis, and mast cells are also an important source of TGF-β in focal tissues. The up-regulation of TGF-β has been identified in fibrotic lesions involving mast cells, such as in scleroderma [50], lymphoedema [46], systemic sclerosis [51] and keloid scars [52].

Mast cells play a very important role in the onset and progression of pulmonary fibrosis, as evidenced by the increased number and activity of mast cells in lung tissue sections. In the lung tissue of IPF patients, the number of interstitial mast cells was 10 times higher than in patients without fibrotic lung disease [53]. Activated mast cells were mainly found near the fibrotic sites and type II alveolar cells [54], and the increase in MCTCs was particularly significant [55]. The degree of pulmonary fibrosis was positively correlated with the number of mast cells [53]. The fibrotic process leads to the activation of mast cells in the lung tissue, with a consequent increase in secretion products. The expression of products secreted by c-kit^+^-activated mast cells, such as trypsin-like enzymes, chymotrypsin-like enzymes and MMPs, were increased in the connective tissue connected to small airways in patients with interstitial lung disease, including IPF [53]. The co-culture of IPF-derived lung mast cells with human lung fibroblasts (HLFs) can activate mast cells, leading to an increase in trypsin-like enzymes and collagen type I, as well as an increase in the proliferation of HLFs [54]. A study of bleomycin (BLM)-induced mice PF models found that, compared to their littermate control mice, mast-cell-deficient WBB6F1-W/W (v) (MCD mice) was protective against PF, while the transplantation of mast cells into the lungs of MCD mice reversed this effect [56].

### 2.4. Eosinophils

Eosinophils are a kind of natural immune cell, accounting for about 5% of leukocytes, which are derived from the proliferation and differentiation of bone marrow hematopoietic stem cells.

It has been reported that an increase in eosinophils in tissues is closely associated with fibrosis. Under the action of inflammatory mediators, active eosinophils induce fibroblasts to produce IL-6 and fibrocytokines, which cause the fibroblasts to proliferate and differentiate into myofibroblasts through autocrine or paracrine secretion, leading to excessive deposition of the ECM in the parenchyma and causing pulmonary fibrosis [57]. The mechanisms of eosinophil activation and migration in patients with IPF are currently unknown. One study found increased expression of IL-5, granulocyte-macrophage colony-stimulating factor (GM-CSF) and regulated on activation of normal T-cell expressed and secreted (RANTES) in the bronchoalveolar lavage fluid (BALF) of patients with IPF, which may be related to the activation of eosinophils and their migration into the lung tissue [58]. Taniguchi et al. [59] found that GM-CSF expression was elevated in the BALF of IPF patients, and suggested that GM-CSF plays an important role in the activation and aggregation of eosinophils in lung tissue. IL-13 expression is upregulated in the BALF and lung tissue from IPF patients, and IL-13 stimulates fibroblast proliferation, cellular matrix protein production and lung fibrosis formation [60]. Acute exacerbation of IPF is the leading cause of death in patients with IPF, and an increased percentage of eosinophils (≥3.21%) in the BALF is a risk factor for the acute exacerbation of IPF [61]. Peterson et al. [62] found that increased eosinophils in the BALF may be a marker of disease progression in IPF. Boomars et al. [63] found that the percentage and absolute number of eosinophils in BALF were negatively correlated with survival in patients with IPF.

### 2.5. Dendritic Cells

Dendritic cells (DCs) originate from bone marrow hematopoietic stem cells, and when mature, they extend many dendritic or pseudopod-like projections protrude, which are then distributed in different forms in various types of tissues, with a predominance in lymphoid tissues [64]. Two main types of mature DCs have been identified: plasmacytoid DCs (pDCs) and myeloid DCs (mDCs) [65]. The function of DCs depends on their maturation state and phenotypic characteristics. The morphology and phenotype of DCs are highly dependent on their environment and the stimuli they receive [66].

Lung DCs are mainly concentrated in the alveolar epithelium and pulmonary interstitium. Pathological biopsies of the lungs have shown that alveolar epithelial hyperplasia in patients with IPF was infiltrated by immature DCs, while fibrotic areas were infiltrated by mature DCs [67]. During the pathogenesis of IPF, immature DCs develop and proliferate from the bone marrow under the regulation of the FMS-like tyrosine kinase-3 ligand (Flt3L), a DC-specific growth factor [68]. Meanwhile, fibroblasts and epithelial cells highly expressed multiple chemokines, leading to an increase in DCs being recruited from the circulation to the lesion site [69]. Lung fibroblasts can influence the progression of IPF by maintaining the number of immature DCs. In addition, DCs with T and B cells in fibrotic lung tissue form abnormal lymphoid tissue. Non-proliferating lymphocytes can induce DC maturation, and it has been shown that DCs and activated non-proliferating lymphocytes are involved in the chronic inflammation caused by interstitial lung fibrosis [67,69,70].

### 2.6. Natural Killer Cells

Natural killer cells (NKs) are innate immune cells in the human body and are an important component of the body’s intrinsic immune response. NKs stimulate DC and B-cell maturation by producing cytokines that support helper T-cell polarization and T-cell activation, thereby coordinating innate and adaptive immune responses [71].

It has been demonstrated that NKs can induce antifibrotic effects in the liver through two independent mechanisms. NKs can prevent fibrosis by directly killing activated hepatic collagen-producing fibroblasts. Moreover, NKs can inhibit hepatic fibrogenesis by releasing soluble antifibrotic mediators, such as interferon-gamma (IFN-γ) [72]. One study found that NKs may have a similar anti-fibrotic function in the lungs [73]. In mice with pulmonary fibrosis that lack NK accumulation and chemokine receptor 3, fibrosis is aggravated due to the lack of INF-γ, while the fibrosis is improved after IFN-γ intervention, which confirms that NKs play an important regulatory role in pulmonary fibrosis through the release of IFN-γ [74].

### 2.7. Natural Killer T Cells

Natural killer T cells (NKTs) are a group of cells that are different from traditional immune cells, as they can recognize antigens like T cells or generate an immune response like NKs in the early stage of disease. Additionally, NKTs can recognize phospholipids and glycolipid antigens presented by the nonclassical antigen-presenting molecule CD1d [75].

The severity of pulmonary fibrosis in NKT-deficient mice was higher than that in control mice. The level of the TGF-β1 in lung tissue was increased after BLM injection and the pulmonary fibrosis of CD1d^−/−^ mice was alleviated after blocking TGF-β1 by neutralizing the monoclonal antibody. IFN-γ was reduced in the lungs of CD1d^−/−^ mice and IFN-γ has been shown to inhibit the production of TGF-β1. These results suggest that IFN-γ-producing NKTs may play a novel anti-fibrotic role in pulmonary fibrosis by regulating the production of TGF-β1 [76].

### 2.8. γδ T Cells

γδ T cells account for approximately 1–10% of human peripheral blood T lymphocytes [77]. Compared to the αβT lymphocytes and B cells, γδ T cells do not express the CD4 and CD8 molecules. They have more unique antigen receptors, and they are major histocompatibility complex (MHC)-unrestricted lymphocytes, which are thought to bridge the gap between innate immunity and adaptive immunity [78,79].

The amount of γδ T cells is increased in the peripheral blood and BALF in patients with IPF [80]. In a mouse model of BLM-induced pulmonary fibrosis, lung γδ T cells could regulate lung fibrosis by inhibiting IL-17A [81]. Simonian et al. also found that γδ T cells inhibit collagen deposition in hypersensitivity pneumonitis, and a subset of these cells represent IL-22. Blocking IL-22 expression exacerbates pulmonary fibrosis [82]. Additionally, γδ T cells may prevent fibrosis by expressing CXC chemokine ligand 10 (CXCL10) [83].

## 3. The Role of Adaptive Immune Cells in IPF

### 3.1. Th1/Th2 Cells

Depending on the type of cytokine produced, CD4^+^ helper cells can be classified as T helper type 1 (Th1) cells, T helper type 2 (Th2) cells, etc. Th1 and Th2 cells secrete multiple cytokines to form a complex cytokine network that regulates the immune response [84]. Th1 cytokines mainly include IL-2, IFN-γ, tumor necrosis factor (TNF), IL-12 and IL-18, whereas Th2 cytokines mainly include IL-4, IL-5, IL-6, IL-10, IL-13 and monocyte chemotactic protein-1 (MCP-1). Th1 cells primarily mediate the cellular immune response. Th1-type immune responses are often associated with inflammation, and an overreaction can cause tissue damage. Th2 cells mainly mediate the humoral immune response, and Th2-type responses are related to the persistence and chronicity of infection, which can inhibit immune inflammation and reduce excessive damage [85].

**Figure 1:**
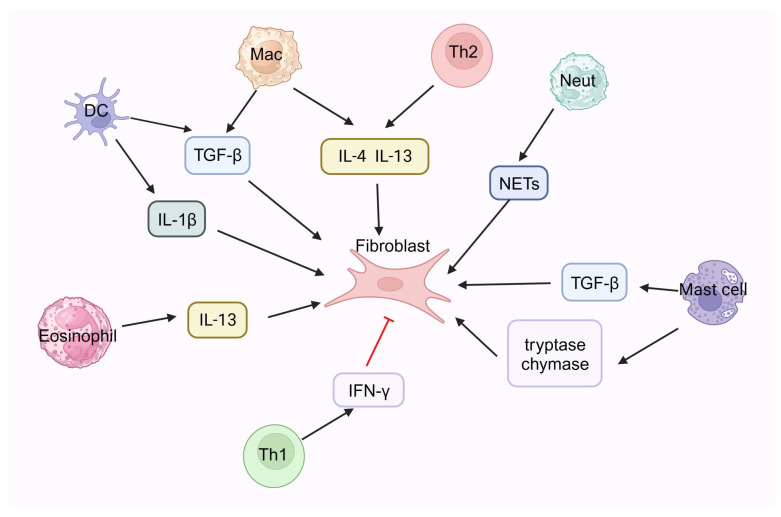
The interaction of immune cells with fibroblast. NETs: neutrophil extracellular traps, TGF-β: transforming growth factor-β, IFN γ: Interferon γ. Created with: “BioRender.com (accessed on 19 October 2023)”.

There is an interactive negative feedback effect between the Th1 and Th2 immune responses, which maintains a normal immune balance. Negative feedback regulation is usually mediated by the production of cytokines. It has been suggested that a Th1/Th2 imbalance may play an important role in the pathogenesis of pulmonary fibrosis [86,87,88]. The process of pulmonary fibrosis may be related to the Th1/Th2 imbalance when there is an overactive pro-fibrotic Th2 response and an underactive anti-fibrotic Th1 response. Th1 cytokines can inhibit the proliferation of fibroblasts and the formation of fibrous tissue, whereas Th2 cytokines can promote the activation and proliferation of fibroblasts, increase collagen synthesis and inhibit its degradation, and ultimately lead to matrix protein deposition and fiber tissue formation (Figure 1). This has been demonstrated in many in vivo and in vitro studies of animal models and patients with pulmonary fibrosis [86,88].

IFN-γ, produced primarily by activated T cells and NKs, plays an important role in the regulation of inflammation and is also a potent anti-fibrotic factor. It inhibits the transformation of Th0 cells into Th2 cells, and inhibits the synthesis and secretion of cytokines such as IL-4 and IL-5 by Th2 cells, thus antagonizing its fibrogenic activity. IFN-γ can significantly inhibit the production of ECM proteins, such as collagen and fibronectin [89]. Chen et al. [90] found that after intratracheal injection of BLM in knockout IFN-γ mice (IFN-γ^−/−^) and wild-type normal mice, the lung parenchymal inflammation, mortality, weight loss and hydroxyproline content in IFN-γ^−/−^ mice were significantly reduced compared with the control group. Intratracheal prophylactic injection of recombinant IFN-γ before an injection of BLM can significantly enhance pulmonary inflammation, suggesting that IFN-γ is involved in the regulation of BLM-induced pulmonary inflammation and fibrosis. IL-12 can induce activated T cells and NKs to secrete IFN-γ. IL-12 has a strong ability to promote Th0 to Th1 cell differentiation, and IFN-γ can inhibit Th2 proliferation by amplifying IL-12-dependent Th1 differentiation. However, IL-4, IL-10 and IL-13 inhibit the proliferation of Th1 cells mainly by down-regulating IL-12 [91]. Keane et al. [92] found that hydroxyproline levels decreased in mice injected with IL-12 compared with control mice injected with human serum albumin, while IFN-γ levels increased in a time-dependent manner in lung tissue and BALF. When anti-IFN-γ antibodies are given at the same time, the anti-fibrotic effect of IL-12 can be weakened.

IL-4 induces the formation and secretion of IL-4, IL-5, IL-6 and IL-10 by Th2 cells and inhibits the formation and secretion of cytokines such as IL-2, IFN-γ and TNF-β by Th1 cells. IL-4 activates fibroblasts to become fibrosecretory active myofibroblasts, and this effect can be attenuated by IFN-γ. Huaux et al. [93] compared BLM-induced lung inflammation and fibrosis in IL-4(^+/+^) and IL-4(^−/−^) mice. They found that the early lung inflammatory response was heavier in IL-4(^−/−^) mice than in IL-4(^+/+^) mice, whereas the degree of lung fibrosis was less severe than in IL-4(^+/+^) mice. However, in the late stage of fibrosis, the degree of fibrosis was more severe in IL-4(^−/−^) mice than in IL-4(^+/+^) mice. Huaux et al. suggested that IL-4 has a dual role in lung injury and fibrosis: in the early phase, IL-4 inhibits T lymphocyte recruitment, while in the late phase, it promotes fibrosis formation. IL-13 is mainly produced by Th2 cells and has the effect of promoting fibrosis. IL-13 has similar biological characteristics to IL-4 and can induce tissue fibrosis through selective stimulation and activation of TGF-β, and its induction effect on fibrosis can be blocked by TGF-β antagonists [94]. Belperio et al. [95] found that neutralizing IL-13 attenuated BLM-induced pulmonary fibrosis in mice. IL-10 is mainly produced by Th2 cells, and it inhibits many cytokines such as IFN-γ, IL-1, TNF, IL-12, CC and CXC chemokines, and can inhibit the function of macrophages. In the inflammatory stage of BLM-induced pulmonary fibrosis, IL-10 can restrict the recruitment of inflammatory cells and the activity of TNF-α, thereby reducing collagen deposition after inflammation [96].

### 3.2. Th9 Cells

Th9 cells are named for their high secretion of the cytokine IL-9. The function of IL-9 is complex, and it provides both beneficial and harmful effects, depending on the disease [97]. Initially, people compared the function of IL-9 with that of IL-4 and found that they have similar roles in the immune response, so IL-9 was considered to be the specific cytokine for Th2 cells [98]. Recent studies have found that IL-9 and IL-4 are basically not derived from the same T cells [99]. Compared to Th2 cells, Th9 cells secrete more IL-9 and less of other cytokines associated with Th2 cells, such as IL-4, IL-5 and IL-13. A corresponding increase in IL-9 levels was found in silicon-induced pulmonary fibrosis mouse models and in patients with IPF [100]. Overexpression of IL-9 in vivo can cause a large amount of collagen and laminin to accumulate in the bronchus, thus producing a pro-fibrotic effect [101].

### 3.3. Th17 Cells

The Th17 cell subpopulation is a group of cells distinct from Th1 cells, Th2 cells and Treg cells; these cells are named Th17 cells because of their high level of IL-17 secretion [55]. The main factor secreted by Th17 cells is IL-17, which plays an irreplaceable role in the occurrence and development of pulmonary fibrosis. Studies have shown that in pulmonary fibrosis, IL-17 presents a high expression level, which is positively correlated with the severity of the disease [79]. Blocking or neutralizing Th17 cell-derived IL-17A can delay the progression of pulmonary fibrosis and promote the rehabilitation of pulmonary fibrosis [102]. IL-17 can increase the expression of IL-6 in respiratory smooth muscle cells induced by TNF-α, and also stimulate the secretion of granulocyte colony-stimulating factor (G-CSF) and macrophage granulocyte colony-stimulating factor (M-CSF) in fibroblasts and respiratory epithelial cells. It can also promote the maturation and activation of neutrophils in various ways, resulting in the aggregation of neutrophils in the lesion. At the same time, it causes damage to alveolar epithelial cells and the alveolar basement membrane [30] and promotes the occurrence of pulmonary fibrosis and other inflammatory reactions. However, studies have shown that Th17 cells do not directly contribute to pulmonary fibrosis [79].

### 3.4. Th22 Cells

Th22 cells are a newly discovered subpopulation of CD4^+^T cells that predominantly secrete IL-22, IL-13, TNF-α and granzyme B [103,104]. Th22 cells express genes for proteins involved in tissue remodeling, such as fibroblast growth factors (FGFs), as well as chemokines involved in angiogenesis and fibrosis, which regulate wound healing, tissue regeneration and fibrosis [105]. The expression of Th22 cells and its related factor IL-22 is significantly higher in IPF patients compared with normal controls, suggesting that Th22 cells are most likely involved in the pathogenesis of IPF and that their role is closely related to IL-22 [106,107].

### 3.5. Cytotoxic T Cells (CTLs, or CD8^+^ T Cells)

CD8^+^ T cells express CD8 surface antigens and play an important role in the body’s anti-virus and anti-tumor immunity. In addition, they are also involved in the development of pulmonary fibrosis. The number of CD8^+^ T cells in the BALF of IPF patients was found to be positively correlated with the degree of pulmonary fibrosis [108]. Activated CD8^+^ T cells secrete IL-13 and mediate BLM-induced pulmonary fibrosis [109]. Furthermore, depletion of CD8^+^ T cells protects mice from fibrotic disease [110].

### 3.6. B Lymphocytes

B lymphocytes are multipotent stem cells derived from bone marrow that can differentiate into plasma cells under antigen stimulation. Plasma cells can synthesize and secrete antibodies, mainly participating in the body’s humoral immunity [111].

Studies have shown that the abnormal activation of B cells is closely related to the formation and progression of lung fibrosis [112]. B cells are required in the development of BLM-induced pulmonary fibrosis, and the depletion of plasma cells reduces the level of BLM-induced fibrosis [113]. IPF patients have high levels of autoimmune antibodies in their peripheral blood targeting B lymphocytes [114]. B-cell activating factor (BAFF) is elevated in the BALF of patients with IPF and in animal models, and BAFF was found to promote pulmonary fibrosis by acting synergistically with IL-1β and IL-17A [115]. Peripheral monocytes from IPF patients can produce more B-cell growth and differentiation factors, compared to healthy individuals. The number of CD20^+^ B cells is significantly higher in the lung tissue of IPF patients, thus producing more IgM and IgA [116].

### 3.7. Regulatory T Cells, (Treg Cells)

Treg cells represent a T-lymphocyte subpopulation exhibiting negative immunoregulatory functions. The transcription factor Foxp3 has a vital role in immune regulation, governing the development and differentiation of Treg cells [117,118,119]. Treg cells are crucial for maintaining immune stability within the body. They achieve this through negative regulation of immunity via immunosuppression and immunocompetence, as well as through the suppression of excessive immune responses [120].

**Figure 2:**
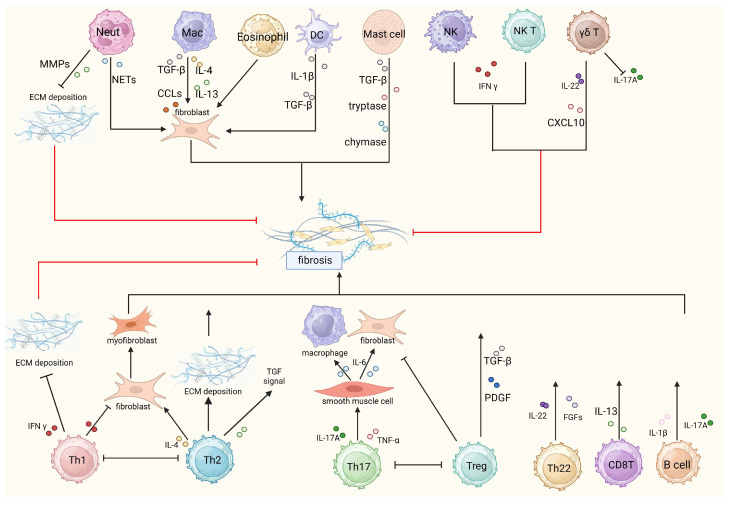
Immune cells in IPF; MMPs: matrix metalloproteinases, ECM: extracellular matrix, NETs: neutrophil extracellular traps, TGF-β: transforming growth factor-β, CCLs: CC chemokine ligands, IFN γ: Interferon γ, PDGF: platelet-derived growth factor, CXCL 10: CXC chemokine ligand 10, TNF-α: tumor necrosis factor α, FGFs: fibroblast growth factors. Created with: “BioRender.com (accessed on 19 October 2023)”.

Autoimmunity and inflammation play an important role in the development of fibrosis, and Treg cells play an irreplaceable role in suppressing the inflammatory response and promoting lung tissue repair. On the one hand, Treg cells can contribute to the progression of pulmonary fibrosis by secreting platelet-derived growth factor (PDGF), TGF-β and other related factors, and by promoting the EMT. On the other hand, Treg cells can inhibit the progression of pulmonary fibrosis by promoting the repair of epithelial cell damage, inhibiting the accumulation of fibroblasts, and suppressing the production and function of relevant pro-inflammatory factors and pro-inflammatory cells [121]. The balance between Treg cells and Th17 cells is associated with pulmonary fibrosis. Th17 cells cause autoimmunity and inflammation, whereas Treg cells suppress inflammation and maintain immune homeostasis [122] (Figure 2). Under normal physiological circumstances, Th17 and Treg cells ought to maintain homeostasis. However, an abundance of Th17 cells combined with a Treg cell deficiency results in the promotion of autoimmunity and tissue damage. This imbalance between pro-inflammatory and anti-inflammatory reactions leads to the worsening of pulmonary fibrosis through the deposition of lung collagen fibers. Studies indicate that the immune balance between Th17 and Treg cells is affected by TCR signaling, costimulatory signaling, cytokine signaling, Foxp3 stability, metabolic processes and microbiota [123]. Some animal experiments have found that the T-cell subsets regulated by Treg cells are different at different stages of pulmonary fibrosis [124]. Treg cells have a pro-fibrotic role during the early disease stages but hinder fibrosis development in the later stages [125]. Therefore, different therapeutic strategies may be required at different stages of the disease.

## 4. Pharmacological Treatment of IPF

The traditional therapeutic drugs for IPF are mainly glucocorticoids and immunosuppressants, but the clinical efficacy observations of traditional therapeutic drugs cannot effectively prevent the development of IPF, and the long-term use of these drugs can produce more side effects. There are only two drugs for IPF that have received FDA approval: pirfenidone and nintedanib. However, they can only delay the progression of symptoms and improve survival to some extent, rather than curing the disease [126,127,128]. Patients may also experience side effects such as nausea, fatigue, and diarrhea when taking these medications [127,129]. At present, the only cure for IPF is lung transplantation. Previous studies have demonstrated the crucial role of the immune response in IPF, and a deeper comprehension of the immune system’s function in IPF may contribute to the development of targeted immunomodulatory therapies to alter the course of the disease.

### 4.1. Pirfenidone

Pirfenidone (PFD) was the first approved drug for the treatment of IPF, which was approved by the FDA in 2014. In 2015, the American Thoracic Society guidelines recommended PFD as one of two drugs for the treatment of IPF [130]. PFD has been used clinically for many years and has shown a positive efficacy, but there are still some side effects in clinical applications, such as photosensitive rash, gastrointestinal symptoms and liver function damage, among others.

It is currently believed that PFD can inhibit the generation of TGF-β, TNF-α, PDGF, connective tissue growth factor (CTGF) and MMPs. PFD exerts its anti-fibrotic effects by inhibiting TGF-β, which strongly promotes ECM deposition and inhibits its degradation; promotes the synthesis of various components of the ECM, as well as fibroblast proliferation and growth; and inhibits the expression of various enzymes and cytokines in the ECM [131]. PFD also reduces the production of TNF-α, thereby inhibiting the inflammatory response, tissue damage and necrosis, as well as subsequent tissue repair and fibrosis [132]. In addition, PFD can reduce the expression of PDGF and others, reduce ECM deposition, stimulate fibroblast mitosis, inhibit collagen synthesis and promote degradation [133]. PFD can also exert anti-inflammatory effects by inhibiting the release of various inflammatory factors, reducing vascular permeability and decreasing the aggregation of inflammatory cells, thereby preventing or slowing down the degree of fibrosis. It has been found that PFD can strongly inhibit the synthesis of TNF-α and IL-6 [132] and can prevent the aggregation of eosinophils and lymphocytes in the airways, significantly reducing the levels of IL-4, IL-5 and IL-13 in the BALF, and thus exerting an anti-inflammatory effect [134]. Furthermore, PFD has the ability to scavenge free radicals, inhibit lipid peroxidation and reduce oxidative stress to exert antioxidant effects [135].

### 4.2. Nintedanib

Nintedanib is a multi-targeted tyrosine kinase inhibitor and the first FDA-approved medicine for the treatment of IPF. Nintedanib can act on IPF-related signaling pathways to inhibit IPF. Nintedanib inhibits PDGF, vascular endothelial growth factor (VEGF), FGF and epidermal growth factor receptor (EGFR), thereby reducing growth factor receptor expression and phosphorylation, and it also inhibits PDGF- and FGF-induced cell proliferation to inhibit fibrosis [136]. Shochet et al. [137] found that nintedanib can inhibit the abnormal activation of EGFR, inhibit the phosphorylation level of Akt downstream of the FGF/EGFR signal, reduce the expressions of collagen I and Smad, and exert anti-fibrotic effects. Ihara et al. [138] showed that nintedanib inhibited EMT by regulating EMT-related gene expression and the TGF-β/Smad pathway, which in turn inhibited pulmonary fibrosis. Furthermore, nintedanib has an effect on cells associated with the promotion of IPF development. It has been found that nintedanib increases the apoptotic clearance of fibroblasts and myofibroblasts by inducing the expression of pro-apoptotic genes in fibroblasts and myofibroblasts to slow down the progression of TGFα-induced pulmonary fibrosis [139]. Nintedanib reduces pulmonary fibrosis by promoting the autophagy of fibroblasts [140], inhibiting mast cell survival [141], altering macrophage polarization to M1 and M2a macrophages [102], and inhibiting neutrophil chemotaxis [142].

### 4.3. Other Pharmacological Therapies for IPF

Over the past few decades, significant progress has been made in understanding the pathogenesis of IPF. The currently accepted pathogenesis is that alveolar epithelial microdamage caused by various factors in vivo and in vitro leads to the abnormal repair of alveolar epithelial cells, and eventually leads to the destruction of the lung structure. As research into the pathogenesis of IPF continues to deepen, a number of new drugs have been proposed, and many of them are now in clinical trials. The following table lists some of the pharmacological therapies in clinical trials (Table 1). Although many of these novel medications have an immune-modulating ability and are promising for certain IPF patients, their precise impact and advantageous effects on the immune response remain elusive. Nonetheless, our limited comprehension of the pathogenesis of IPF, particularly regarding the interplay between current innovative treatments and the immune system, poses a significant obstacle to achieving future breakthroughs in precision medicine.

## 5. Conclusions

IPF is a complex, aging-related disease that is highly aggressive and lethal, but the treatment options and medications available for this disease are very limited. Although we have a certain understanding of immune cells in the pathogenesis of IPF, and the severity of IPF can be significantly improved by modulating the pulmonary immune system, many difficulties remain unsolved. The immune system is a large and complex system, and the occurrence and development of IPF involves the joint participation of a variety of immune cells. With further research, we have found that the roles of immune cells in IPF are not simple, and they tend to exhibit both favorable and harmful effects. Yet most of our research is still based on a single understanding of the causal relationship of the disease, which is not in line with the actual situation of the disease. Pharmacological treatments are not as effective as they could be, and lung transplantation remains the only truly effective treatment option available. In order to develop more effective treatments, the role of the immune system in the development of pulmonary fibrosis needs to be explored in detail to find more effective potential molecular targets that could open up a new chapter in the pharmacological treatment of IPF.

## Figures and Tables

**Table 1:** Ongoing clinical trials in idiopathic pulmonary fibrosis.

Drug	Target	Mechanism	Phase
Treprostinil	PGI2 receptor	PGI2 receptor agonist	III
Acetylcysteine	Free radical	Free radical inhibitor	III
Axatilimab	CSF-1R	CSF-1R antagonist	III
Zinpentraxin alfa	APCs	APCs modulator	III
BI-1015550	PDE4B	PDE4B inhibitor	III
BMS-986278	LPAR1	LPAR1 inhibitor	III
Pamrevlumab	CTGF	CTGF inhibitor	III
lanalumab	BAFF-R	BAFF-R inhibitor	III
Belumosudil	ROCK1 + ROCK2	ROCK1inhibitor, ROCK2 inhibitor	II
Ifenprodil Tartrate	NMDA receptor	NMDA receptor inhibitor	II
Nalbuphine Hydrochloride	Κ opioid receptor + μ opioid receptor	κ opioid receptor agonist, μ opioid receptor inhibitor	II
Jaktinib hydrochloride	JAK1 + JAK2 + JAK3	JAK1 inhibitor, JAK2 inhibitor, JAK3 inhibitor	II
Sodium Pyruvate	IL-6 + Reactive oxygen species	IL-6 inhibitor, Reactive oxygen inhibitor	II
(68Ga) CBP8	collagen I	collagen I modulator	II
Garadacimab	F12	F12 inhibitor	II
Pamufetinib	VEGFR + c-Met	VEGFR antagonist, c-Met inhibitor	II
Saracatinib	FYN + SRC family	FYN inhibitor, SRC family inhibitor	II
TDI-01	ROCK2	ROCR2 inhibitor	II
Romilkimab	IL-13 + IL-4	IL-13 inhibitor, IL-4 inhibitor	II
CM-101	CCL24	CCL24 inhibitor	II
IDL-2965	integrin αvβ1 + integrin αvβ3 + integrin αvβ6	integrin αvβ1 inhibitor, integrin αvβ3 antagonist, integrin αvβ6 antagonist	II
GRI-0621	RARβ2 + RARγ	RARβ2 agonist, RARγ agonist, NK cell receptor antagonist	II
Vixarelimab	IL-31R-β (OSMRβ)	OSMRβ inhibitor	II
RVT-1601		mast cell stabilizer	II
Pamapimod	p38 MAPK	p38 MAPK inhibitor	II
Yinfenidone Hydrochloride	TGF-α + TGF-β	TGF-α inhibitor, TGF-β inhibitor	II
Leramistat	TNF	TNF inhibitor	II
C-188-9	STAT3	STAT3 inhibitor	II
Famitinib Malate	FGFRs + PDGFR + VEGFR + VEGFR2 + c-Kit	FGFR antagonist, PDGFR inhibitor, VEGFR antagonist, VEGFR2 antagonist, c-kit inhibitor	I
Setogepram	GPR40 + GPR84	GPR40 agonist, GPR84 inhibitor, autophagy agonist	I
BAY-85-8102	translocator protein(TSPO)	TSPO inhibitor	I
Deupirfenidone	CCL2 + IL-10 + IL-6 + IL-1 + TNF-α	CCL2 inhibitor, IL-10 inhibitor, IL-6 inhibitor, IL-1 inhibitor, TNF-α inhibitor	I
ANG-3070	DDR1 + DDR2 + PDGFRα + PDGFRβ	DDR1 inhibitor, DDR2 inhibitor, PDGFRα inhibitor, PDGFRβ inhibitor	I

## Data Availability

Not applicable.
